# The Importance of Vitamin D and Magnesium in Athletes

**DOI:** 10.3390/nu17101655

**Published:** 2025-05-13

**Authors:** Ligia J. Dominguez, Nicola Veronese, Francesco Saverio Ragusa, Salvatore Maria Baio, Francesco Sgrò, Arcangelo Russo, Giuseppe Battaglia, Antonino Bianco, Mario Barbagallo

**Affiliations:** 1 Department of Medicine and Surgery, “Kore” University of Enna, 94100 Enna, Italy; arcangelo.russo@unikore.it; 2 Geriatric Unit, Department of Internal Medicine and Geriatrics, University of Palermo, 90100 Palermo, Italy or nicola.veronese@unicamillus.org (N.V.); francescosaverioragusa@gmail.com (F.S.R.); salvatoremariabaio@gmail.com (S.M.B.); mario.barbagallo@unipa.it (M.B.); 3 Faculty of Medicine, Saint Camillus International University of Health Sciences, 00131 Rome, Italy; 4 Department of Human and Society Sciences, “Kore” University of Enna, 94100 Enna, Italy; francesco.sgro@unikore.it; 5 Sport and Exercise Sciences Research Unit, Department of Psychology, Educational Science and Human Movement, University of Palermo, 90133 Palermo, Italy; giuseppe.battaglia@unipa.it (G.B.); antonino.bianco@unipa.it (A.B.); 6 Regional Sports School of Italian National Olympic Committee (CONI) Sicilia, 90141 Palermo, Italy

**Keywords:** vitamin D, magnesium, athlete, muscle, bone, ATP, cardiovascular, respiratory

## Abstract

Vitamin D and magnesium are essential nutrients that play key roles in an athlete’s performance, recovery, and overall health. Vitamin D is crucial for bone health (aiding calcium absorption and preventing stress fractures), muscle function (preventing weakness and injury), and reducing respiratory infections. Magnesium is fundamental in muscle function, adenosine triphosphate production for muscle contraction, electrolyte balance, bone strength, and cardiovascular health. The magnesium requirement of healthy adults is estimated at 300–400 mg/day, but there is evidence that athletes may have higher magnesium needs compared to sedentary persons. Magnesium and vitamin D are closely linked—vitamin D aids magnesium absorption, while magnesium is vital for vitamin D synthesis, transport, and activation. Given their importance in athletes, this article explores their functions, interactions, and the effects of deficiencies and supplementation in athletic populations.

## 1. Introduction

Proper nutrition is crucial for athletes to enhance their performance during training and competitions and maintain internal balance. A variety of foods and supplements is needed to meet nutritional needs [1,2]. Adequate nutrition provides energy, supports body structure, and supplies essential vitamins and minerals that regulate metabolic processes. However, despite sufficient intake, athletes may still face nutrient deficiencies, which can hinder performance and increase injury risk [2,3]. Magnesium and vitamin D are key nutrients that are essential for athletic performance, recovery, and overall health.

Once known mainly for its role in calcium balance, vitamin D is now recognized as a hormone with wide-ranging effects on immunity, protein and hormone synthesis, gene signaling, and cell regeneration [4,5]. Primarily synthesized through sun exposure, smaller amounts come from foods like oily fish, egg yolks, and dairy [6]. Its importance in sports nutrition is increasingly acknowledged, mainly due to the high prevalence of vitamin D deficiency across many sports, which has been linked to reduced strength, endurance, and greater injury risk [7,8,9]. Studies show that 32% of professional basketball players are deficient and 47% have insufficient 25(OH)D levels. In the National Football League (NFL), 26% are deficient and 42–80% have insufficient levels. Similar trends are seen in swimmers, dancers, taekwondo fighters, volleyball players, weightlifters, runners, and others [10].

Magnesium is an essential mineral involved in over 600 enzymatic reactions [11], including energy metabolism [11,12,13], cell growth [14,15], glycolysis [16,17], and protein synthesis [18,19]. As Mg^2+^, it forms the Mg-ATP complex [11,12,18,20]—vital for energy production and key physiological functions like muscle contraction [21,22,23], blood pressure regulation [24,25], and nerve conduction [26]. Magnesium is crucial for muscle function, supporting energy metabolism and proper contraction/relaxation [27,28,29,30]. Higher serum magnesium levels are linked to better muscle performance in both older adults [22,31] and athletes [32,33]. Deficiency may impair neuromuscular function and contribute to cramps, [34,35] although evidence on its role in exercise-induced cramps remains limited [36].

Magnesium and vitamin D are closely interconnected—vitamin D supports the absorption of magnesium, while magnesium plays a critical role in the synthesis, transport, and activation of vitamin D [37,38]. Because magnesium and vitamin D are essential in athletes, this article will discuss their overall functions, their interaction, and the implications of their deficiencies and supplementation in athletes. Although there are reviews and meta-analyses on vitamin D and magnesium in athletes, the available studies are small and inconclusive. Therefore, we aim to raise interest in developing future more powerful studies about these essential nutrients in the athletes.

For the present article, we performed a search of the literature published in PubMed, from database inception to 1 March 2025, utilizing terms such as “vitamin D” or “magnesium” and “athlete” or “systematic review” or “meta-analysis” or “umbrella” or “randomized controlled trial” or “RCT” or “trial”. We enhanced the search by incorporating studies known to the authors and conducting additional forward citation searches. We excluded other types of articles, such as editorials, comments, letters to the editor, case reports, case series, short communications, short reports, and perspectives.

## 2. Vitamin D in Athletes

Despite assumptions that outdoor athletes have adequate vitamin D, recent studies show their levels are similar to the general population. Factors like geography and sport type affect vitamin D status. Deficiency can lead to higher morbidity and conditions like osteomalacia, osteoporosis, and other extraskeletal diseases.

### 2.1. Synthesis, Sources, Metabolism, and Assessment

Since Nobel laureate Windaus identified vitamin D’s structure in 1930 [39], research has evolved from focusing on bone metabolism to broader health areas. The discovery of 25(OH)D in 1968 [40,41] and 1,25(OH)2D [42,43] expanded its study to immune diseases, infections, cardiovascular issues, and cancer [44,45]. Vitamin D regulates the immune system, influences T lymphocytes, cytokines, and apoptosis [4,46], and promotes phosphate and magnesium absorption while preventing renal excretion. Its functions extend far beyond bone health.

Vitamin D is primarily synthesized in the skin, with dietary intake playing a smaller role due to limited daily consumption of vitamin D-rich foods, even among athletes. Table 1 lists dietary sources of vitamin D. Supplements may be needed for those at risk of deficiency, including athletes.

Cutaneous synthesis of vitamin D3 occurs from 7-dehydrocholesterol upon UV exposure (290–320 nm) [44]; UV exposure equivalent to 25% of the minimum erythematic dose (MED) generates 25 µg (1000 IU) [47], while 15 min of midday sun (∼1 MED) produces 250 µg (10,000 IU) [48]. Face, hand, and arm exposure to one-third to one-sixth of MED yields 200–600 IU of vitamin D [49]. Factors like age, season, skin color, latitude, and sunscreen use affect synthesis efficiency [50,51,52].

After entering circulation, vitamin D_2_ (ergocalciferol) and D_3_ (cholecalciferol) bind weakly to their binding protein (DBP) for transport and are stored in fat. In the liver, they are converted to 25(OH)D—the main circulating form—by CYP2R1 and CYP27A1, with additional conversion possible in other tissues [53]. High doses may slow this process [54]. In the kidneys, CYP27B1 forms active 1,25(OH)_2_D, while CYP24A1 produces inactive 24,25(OH)_2_D [55] (see Table 2 for details). The active form regulates functions via vitamin D receptor (VDR) [53], discovered in 1969 [56], and cloned in 1987 [57,58]. VDR is essential for vitamin D function, as shown in knockout models [59], and is widely present in human tissues [60,61].

A recent study using a food frequency and lifestyle questionnaire (FFLQ) in athletes found no link between vitamin D intake and serum 25(OH)D levels. However, strong correlations were observed with tanning bed use (spring), supplement intake (fall), and BMI year-round, suggesting non-dietary factors significantly influence vitamin D status [62].

Serum 25(OH)D is the best accepted marker of vitamin D status and is used to assess deficiency and guide intake recommendations [63]. By contrast, 1,25(OH)_2_D is tightly regulated and levels may remain normal in deficiency due to elevated parathyroid hormone (PTH) levels, which stimulates its production. Optimal vitamin D keeps PTH within normal limits [6]. An international consensus defined vitamin D status as sufficient >20 ng/mL, insufficient 12–20 ng/mL, deficient <12 ng/mL, and potential toxicity >100 ng/mL with high calcium intake [64].

### 2.2. Vitamin D Deficiency in Athletes

Vitamin D deficiency is common across sports, with 32% of professional basketball players and 26% of NFL players being deficient; 42–80% showed insufficiency. Similar trends appear in swimmers, dancers, weightlifters, and others [10]. Dark-skinned athletes need up to 10 times more UVB to produce adequate vitamin D. By contrast, a study of mostly Caucasian hockey players showed no deficiency and only 13% showed insufficiency [65]. Athletes living north of 35° N (e.g., Finland, Russia) face reduced UV exposure, especially in winter, limiting vitamin D synthesis [66]. In Finland (60° N), over 80% of athletes had low 25(OH)D levels [67]. Among Russian youth soccer players, 42.8% had below 30 ng/mL, but 5000 IU/day of cholecalciferol for two months raised levels to 30–60 ng/mL in 74% [68].

A recent meta-analysis found outdoor athletes had slightly higher vitamin D levels than indoor athletes, with a crude difference of 3.73 ng/mL—significant only in Asian groups. After adjusting for season, latitude, and race, indoor athletes had 4.45 ng/mL lower levels. However, training type alone had minimal impact, suggesting it should not solely guide supplementation decisions [69]. Another meta-analysis of 23 studies (2313 athletes, mean age 22.5) found that 56% had low 25(OH)D (<32 ng/mL) levels, especially in winter/spring, indoor/mixed sports, and at latitudes >40° N [70]. Supplementation improves vitamin D levels but the effects on muscle performance are mixed [71]. As such, a meta-analysis of 13 RCTs (433 athletes) showed 3000–5000 IU/day raised serum levels, especially in winter and >45° N, but had no clear effect on performance after 12 weeks—likely due to trial heterogeneity [72]. More targeted RCTs are needed. However, it is important to clarify that vitamin D is necessary to maintain muscle mass and function but does not have anabolic effects.

In elite Para-athletes, a systematic review of 10 studies (*n* = 355; 69.6% male) found that 43.2% showed insufficiency and 28.1% had deficient vitamin D levels, with higher insufficiency in winter (74.1%) compared to summer (57.1%), which is still remarkably high. Wheelchair athletes in indoor sports were especially at risk [73]. Ongoing education and preventive strategies are necessary for these athletes.

Physical exercise can boost vitamin D levels. Studies show that regular exercise or high activity is linked to higher serum 25(OH)D levels, even with limited sun exposure [74,75,76]. One study found this effect in both summer and winter [77]. Sun et al. showed that 30 min of cycling at 70% VO_2_max significantly raised 25(OH)D levels, especially in men [78]. Similar increases were seen after stretch-shortening cycle exercises [79]. However, some studies found no acute changes, possibly due to timing issues [80,81].

Thus, routine vitamin D testing and targeted supplementation—especially in winter, low-UVB regions, and/or indoor athletes—are key to supporting performance, maintaining athlete health, and preventing issues like stress fractures. Adequate levels may offer broader health benefits, but supplementation should be individualized to avoid excessive dosing and potential risks.

### 2.3. Effects of Vitamin D of Particular Interest for Athletes

Vitamin D affects more than bone health due to VDRs in many tissues—skin, muscle, immune cells, brain, and fat, among others [4,5,60,61,82]. VDR activation by 1,25(OH)_2_D triggers genomic and non-genomic effects, including immune modulation, muscle regulation, anti-cancer activity, and potential benefits for cardiovascular and metabolic health—many of which are especially relevant to athletes [82].

#### 2.3.1. Muscle

Muscle weakness is a common, though non-specific, symptom of vitamin D deficiency [6], highlighting its importance in athletic performance [83]. Calcitriol through the VDR in muscle tissue promotes cell growth and type II fiber development, enhancing strength and speed [83,84,85,86,87,88,89]. Though early debate questioned VDR presence in muscle [85,86], later studies confirmed it [87]. While some research links vitamin D to better muscle performance, the results are mixed, possibly due to varying deficiency levels and response to supplementation [71]. Still, studies showed improved type II fiber size after treatment [90], and that higher pre-exercise 25(OH)D levels may reduce post-exercise muscle weakness and aid recovery [79].

**Figure 1:**
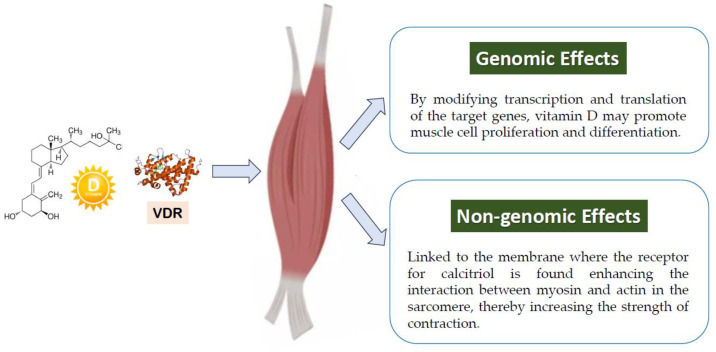
Genomic and non-genomic effects of vitamin D on muscle through VDR (vitamin D receptor).

Vitamin D’s direct effects on muscle have been mainly studied in preclinical models. In skeletal muscle, 1,25(OH)_2_D binds to VDR, initiating genomic and nongenomic responses (Figure 1). Genomically, it forms a VDR-RXR complex that regulates gene expression via VDRE. Nongenomically, VDR localized to the membrane triggers rapid signaling (e.g., c-Src, MAPK) and enhances myosin–actin interaction, enhancing muscle contraction, proteostasis, and calcium homeostasis [5,53]. High doses of 1,25(OH)_2_D can inhibit myoblast proliferation but promote hypertrophy in mature myotubes. In C2C12 muscle cells, 1,25(OH)_2_D_3_ increases myogenin expression through a VDRE on its promoter, resulting in larger myotubes and elevated myosin heavy chain expression, underscoring its role in muscle development [91].

Whole-body and tissue-specific VDR knockout models show decreased grip strength and muscle fiber size, alongside increased atrophy-related gene expression, underscoring VDR’s role in muscle integrity. Post-injury vitamin D enhances regeneration by promoting cell proliferation, reducing apoptosis, and aiding repair [92]. Vitamin D also modulates calcium transport via nongenomic actions, facilitating contraction, while VDR activation regulates calcium flow, further supporting muscle function [93].

Therefore, an optimal level of vitamin D appears essential for athletes to maintain muscle health and aid in injury repair, but there are few studies specifically focused on these actions. There is considerable room for further study.

#### 2.3.2. Bone

Through VDR activation by 1,25(OH)_2_D, vitamin D enhances calcium, phosphate, and magnesium absorption, supports bone mineralization, and stimulates osteocalcin [5,44]. Alongside parathyroid hormone (PTH), it promotes bone resorption via receptor activator of nuclear factor kappa-Β ligand (RANKL) signaling. Vitamin D also inhibits PTH and induces FGF23, forming a feedback loop that maintains calcium–phosphate balance and supports bone growth [6,94].

An observational study of NFL players found lower vitamin D levels in those with fractures, independent of career length [95]. A 2015 RCT in young male jockeys showed that vitamin D supplementation improved bone properties—such as cortical content, density, and bone area—within six months [96].

Bone stress injuries make up ~20% of sports medicine cases [97] and are common among athletes and military recruits, often linked to pain, performance loss, and training time missed [98]. Nearly one-third of runners [99] and 21.1% of track athletes report stress fractures [100]. There is evidence suggesting that vitamin D could play a role in preventing stress fractures in athletes [83]. Risk factors include sudden training increases, low bone mineral density (BMD), menstrual irregularities, and low vitamin D levels. Serum 25(OH)D levels below 20 ng/mL are associated with higher stress fracture risk [101], while higher levels support faster recovery [102].

A large RCT in U.S. Navy women (*n* = 3700) showed that daily vitamin D and calcium supplementation reduced stress fractures by 20% [103]. However, data on elite athletes remain limited due to complex influencing factors (i.e., age, overtraining, poor diet, smoking, and potential menstrual disorders). A meta-analysis by Dao et al. including 8 studies found significantly lower 25(OH)D levels in military personnel with stress fractures [104].

Shimasaki et al. reported that Japanese male soccer players with 25(OH)D levels below 30 ng/mL had a higher risk of fifth metatarsal stress fractures [105]. In a two-year study, Nieves et al. found that vitamin D intake in young female runners was associated with higher hip BMD and fewer stress fractures [106].

Thus, the usual bone mineralization actions of vitamin D are necessary in athletes, and its deficiency, frequent in this population, may be related to the increased risk of stress fractures, even in this young population.

#### 2.3.3. Cardiovascular and Respiratory Systems

Intense training, especially in running sports, induces cardiovascular adaptations that may mimic pathology but reflect well-adapted organs [107]. While vitamin D’s role in cardiovascular disease is debated despite extensive research [108,109], evidence from animal and human studies shows that deficiency negatively affects heart structure and function. In athletes, low vitamin D levels are linked to reduced cardiac hypertrophy following training [110,111].

Vitamin D and its binding protein are vital for lung function. Deficiency is linked to respiratory conditions like asthma, infections, and chronic obstructive pulmonary disease [6]. College athletes with low vitamin D levels face increased risk of acute upper respiratory infections (URTIs) [112].

Maintaining vitamin D levels above 38 ng/mL helps to protect against viral infections; levels below this double the risk of URTIs [113]. In athletes, levels above 30 ng/mL support immunity and reduce URTI risk [114]. Supplementing with 4000 IU/day is effective in high-risk groups [115], and adequate levels are linked to lower influenza A rates [116] and were shown to potentially reduce COVID-19 mortality [4,117]. A meta-analysis of 25 RCTs found that vitamin D lowers respiratory infection risk, especially with daily/weekly dosing and in individuals with very low (<10 ng/mL) baseline levels (−70% risk) [118]. This is particularly relevant in athletes who frequently have vitamin D deficiency [10,65,66,67,68] and increased risk of infections.

Athletes have higher rates of asthma and allergic rhinitis than the general population. Low vitamin D levels are associated with elevated eosinophils, IgE, severe asthma, and increased hospitalizations [114]. Screening athletes and providing vitamin D supplementation may help to reduce asthma flare-ups and URTIs.

Thus, the role of vitamin D in cardiac function in athletes is not well defined. From a respiratory perspective, it is essential to prevent and treat vitamin D deficiency due to the increased risk of asthma and respiratory infections in this population.

## 3. Magnesium in Athletes

Magnesium, essential in all living cells, acts as a key cofactor for ATP—the main source of cellular energy—to which it is closely associated [11,16,25,119,120]. Its roles in energy production, muscle function, and glucose regulation make it a potential performance mediator and enhancer for athletes.

### 3.1. The Importance of Magnesium in Cellular Function

Magnesium is vital for numerous cellular functions, binding to nucleotides and regulating over 600 enzymatic reactions, including those for DNA/RNA processes and all ATPase reactions [11,16,25,121] (Table 3). It supports glucose, lipid, and protein metabolism [122,123,124], neuromuscular and cardiac function, hormone release, and CNS signaling [24,125,126,127]. Furthermore, it functions as a secondary messenger in intracellular signaling pathways [125,128,129,130], regulating circadian rhythm [131] and ion transport [132].

Magnesium is 98% intracellular, with only 2% in serum, which is tightly regulated by intake, absorption, renal excretion, bone storage, and tissue demand [16,25,133]. Magnesium-rich foods are primarily plant based (Table 4).

Many in Europe and the U.S. have low magnesium intake due to processed diets [134,135,136,137,138]. Deficiency, even mild, is linked to obesity, diabetes, hypertension, and other chronic diseases via inflammation and oxidative stress [11,16,17,24,119,122]. Severe deficiency (hypomagnesemia) (serum levels <0.75 mmol/L, <1.7 mg/dL) causes nonspecific symptoms like fatigue and cramps [11] and is more common in people with type 2 diabetes and hospitalized patients [139]. It is associated with higher all-cause and cardiovascular mortality [139,140]. Other people for which magnesium deficiency is particularly common are athletes, as will be discussed below.

### 3.2. Magnesium Deficiency in Athletes

Exercise influences magnesium use and distribution, while magnesium supports strength and cardiorespiratory function [11,24,141]. In response to exercise, magnesium is transported to areas where energy production occurs [142]. During prolonged activity, magnesium shifts to muscles to aid energy production; short-term exercise may raise serum levels due to reduced plasma volume [141]. Endurance exercise can cause magnesium loss through sweat, increasing deficiency risk and arrhythmia [143] while increasing magnesium requirements [29]. A study in well-hydrated endurance athletes found no plasma magnesium changes during incremental cycling, emphasizing plasma volume and hydration as key factors in magnesium balance during exercise [144]. Conversely, a study by Siquier-Coll et al. assessed magnesium and phosphorus level changes post-exercise in normothermic (22 °C) and hyperthermic (42 °C) conditions, reporting that hyperthermic exercise lowered magnesium levels but not phosphorus levels. Heat acclimation reduced magnesium loss in sweat but increased urinary excretion, with the authors concluding that magnesium supplementation is advisable for athletes in hot conditions [145].

Regular exercise benefits physical fitness [146], mental health [147,148], and chronic disease prevention [149,150]. Nutrient availability, including magnesium, is key for muscle adaptations to endurance and resistance training [151,152,153,154,155]. Magnesium supports nerve function, muscle contraction [125,156,157], energy metabolism, the immune response, and reduces oxidative stress [11,37,142,158]. Intense exercise increases magnesium needs due to inflammation and muscle damage [159]. Athletes may have higher magnesium requirements compared to sedentary individuals [29,160]. Deficiency can impair performance [161] and cause issues like oxidative damage [162], arrhythmias [163], muscle weakness [29], and hypomagnesemia [164]. Athletes may require supplementation when dietary intake is insufficient.

Blood magnesium levels vary with exercise intensity and duration—rising briefly after short, intense activity and dropping after prolonged endurance exercise, often due to sweat loss [29,165]. Studies show that many athletes, including ultra-endurance racers, rugby players, and elite female gymnasts, fail to meet recommended magnesium intake levels, highlighting a widespread risk of deficiency among active individuals [166,167,168,169,170]. Czaja et al. found that elite Polish runners consumed 256 ± 111 mg (females) and 284 ± 58 mg (males) of magnesium daily. However, diet analysis software overestimated intake by 159–181%, indicating that food records may not reliably reflect actual magnesium intake [168].

Thus, physically active persons are at greater risk of magnesium deficiency due to increased demands; hence, they may require more magnesium than inactive people [29,30,171,172]. A systematic review of 14 studies found that athletes had lower serum magnesium levels despite higher intake and urinary excretion, suggesting higher needs [173]. In addition, a meta-analysis of 31 studies showed that most athletes consumed less than the RDA [174]. Pollock et al. found that 22% of elite British Olympic and Paralympic athletes had intracellular magnesium deficiency, especially among women, those of Black/Mixed ethnicity, and those with Achilles/patella tendon pain [175].

Therefore, it is necessary to ensure that magnesium levels are sufficient in the diet of athletes and supplementation may be necessary, especially in high-performance athletes, due to the greater consumption and therefore greater requirements for this fundamental nutrient.

### 3.3. Effects of Magnesium of Particular Interest for Athletes

Exercise and magnesium have a reciprocal relationship—exercise regulates magnesium distribution for energy needs, while magnesium supports muscle strength, cardiovascular health, bone integrity, and immunity.

#### 3.3.1. Muscle

Magnesium is essential for skeletal muscle function, supporting energy production, protein synthesis, oxidative phosphorylation, and glycolysis [11,119] (Table 5). It influences neuromuscular function, cell signaling, bone development, cell proliferation, and genomic stability [11,176]. Magnesium supports ion transport, muscle contraction, neuron excitability, and cellular ion balance [119,177]. Its high solubility, small ionic radius, and strong hydratability make it highly stable and larger than other cations (i.e., calcium, potassium, and sodium). This limits its ability to pass through narrow channels, requiring energy-consuming dehydration—explaining its role as a natural calcium channel blocker [178,179].

**Figure 2:**
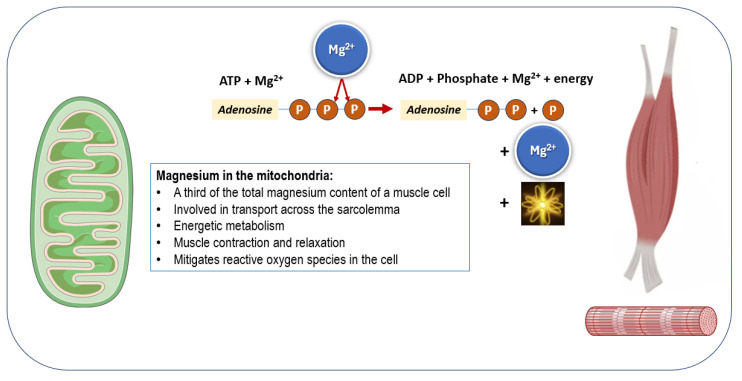
Magnesium is crucial for energy production, as ATP cannot be activated until it binds with magnesium. The ability of muscle to maintain its available energy during alterations in workload is critical for normal function. Magnesium plays a role in sarcolemma transport, energy metabolism, and the processes of muscle contraction and relaxation. Additionally, magnesium helps to reduce oxidative stress (reactive oxygen species). ADP: adenosine diphosphate; ATP: adenosine triphosphate.

About one-third of muscle magnesium is in mitochondria, which are essential for energy metabolism [180]. Magnesium supports mitochondrial function, with deficiency leading to reduced efficiency, increased oxidative stress, and reduced aerobic capacity in muscle [180,181]. Thus, it is essential for energy metabolism and mitochondrial health. Magnesium deficiency impairs mitochondrial efficiency, increases reactive oxygen species production, and contributes to muscle decline [11,22]. It activates key glycolytic enzymes [17,182] and forms Mg-ATP, the biologically active ATP form that is vital for muscle contraction and enzyme function [180]. In fact, most ATP exists as Mg-ATP, the active form needed for enzymatic activity and energy production [11,176]. A summary of the role of magnesium in mitochondria is shown in Figure 2.

Magnesium supplementation has shown mixed results in improving athletic performance. Positive outcomes were noted in recreational athletes [183] and elite soccer players [184], but most RCTs found no improvements in muscle strength or performance in volleyball players [185], triathletes [186], cyclists [187], physically active men [188] and women [189], recreational runners [190], or marathon runners [191]. However, these studies often did not assess magnesium status or relied on plasma/serum measurements, which are less sensitive to change.

Variability in supplementation outcomes may stem from differences in magnesium status at baseline, dosing, duration (10 days to 32 weeks), and salt form used (e.g., citrate, oxide, lactate, sulfate, bisglycinate chelate, and magnesium creatine chelate). Most studies focused on young adults, understandably because most athletes or physically active persons are young. However, an example of magnesium’s benefit for muscle function is its role in counteracting age-related decline. In the InCHIANTI study, higher magnesium levels were linked to better muscle performance [22]. In addition, Veronese et al. found that 12 weeks of supplementation (300 mg/day) improved physical function in older women, especially those with dietary intakes below the RDA [31].

Thus, there is little doubt that magnesium is essential for muscle function and that its deficiency, likely in athletes with higher requirements, should be avoided and corrected.

#### 3.3.2. Bone

Fragility fractures pose a major public health issue, impacting health, quality of life, and increasing societal costs [192,193]. While calcium and vitamin D are well studied, other dietary components can contribute [194]. As such, magnesium plays a role as well, and its deficiency is linked to increased osteoclast activity and reduced osteoblast function [195,196,197,198], which may be improved with supplementation [196,197,199,200,201].

Several studies link higher dietary magnesium intake to improved BMD [202,203,204,205] and possibly reduced fracture risk [204,206], although fracture data are less conclusive due to limited study quality. A recent meta-analysis by our group including four high-quality studies (119,755 participants) found that low serum magnesium levels are strongly associated with increased fracture risk [207], underscoring magnesium’s importance in bone health and its potential as a modifiable risk factor, especially in at-risk groups like athletes.

Magnesium deficiency has been associated with lower BMD. In a study of elite swimmers, Matias et al. found that magnesium intake was below recommended levels and strongly predicted BMD, even after adjusting for relevant confounders (i.e., energy, calcium, vitamin D, and phosphorus intake) [208], highlighting its importance for bone health in young athletes in low-impact sports.

Although there is evidence from preclinical studies of the importance of magnesium in bone health, there is no definitive evidence of its role in fracture prevention, and very few studies have been conducted in athletes. Hence, there is ample room for future research.

#### 3.3.3. Cardiovascular System

Magnesium imbalance is linked to cardiovascular issues [24,119,140]. Low magnesium levels increase arrhythmia risk (i.e., atrial fibrillation, torsades de pointes, and long QT syndrome) [11,119,209] and contribute to vascular problems like endothelial dysfunction and hypertension [24]. In pregnancy-related hypertension (preeclampsia/eclampsia), intravenous magnesium helps to reduce complications via calcium channel blockade, vasodilation, and other mechanisms such as lowering oxidative stress and regulating Transient Receptor Potential Cation Channel Subfamily M Member (TRPM) 6 and TRPM7 [24,210,211] (Table 6).

Magnesium acts like an antithrombotic by inhibiting platelet aggregation [212,213], while its deficiency triggers oxidative stress and nuclear factor kappa-light-chain-enhancer of activated B cells (NF-κB)–mediated inflammation, increasing cytokine production and promoting atherosclerosis, thrombosis, and vascular calcification [11,119,212,214]. It also stabilizes membranes by blocking calcium channels [215] and regulating potassium, which contributes to antiarrhythmic effects. Strong evidence links even mild deficiency to cardiovascular disease [216], making inadequate magnesium intake a frequent public health concern (Table 4).

While physical activity offers many benefits, athletes can still develop heart disease. Celeski et al. found that high exercise levels may be associated with higher coronary artery calcium scores and atherosclerotic plaque burden, especially calcified types, challenging the assumption they are harmless [217]. More large-scale studies are needed to understand the risks in athletes.

Elite athletes may be more prone to hypomagnesemia, which has been linked to a higher risk of sudden death from arrhythmias [218]—more common in athletes than in the general population [219]. Stendig and Lindberg linked sudden death in athletes during exertion to chronic magnesium deficiency [220], a connection first reported by Chadda et al. [221]. Magnesium loss through sweat in endurance running can lead to severe deficiency and increase the risk of serious arrhythmias [143,163].

Correcting magnesium levels helps to control ventricular response in atrial fibrillation, reduce ventricular extrasystoles, and prevent torsades de pointes. It may also benefit polymorphic and supraventricular tachycardia [163]. Magnesium deficiency increases arrhythmia risk through calcium overload, electrolyte imbalance, impaired Na^+^/K^+^-ATPase activity, and inflammation-related cardiac effects [163,218]. A study of 88,375 women from the Nurses’ Health Study found that higher dietary and plasma magnesium levels were linked to significantly lower sudden cardiac death (SCD) risk. Over 26 years, 505 SCD cases occurred, with a 37% lower risk in participants with high dietary magnesium levels and 77% lower risk with high plasma levels. Each 0.25 mg/dL increase in plasma magnesium level reduced the SCD risk by 41% [222].

In the Rotterdam Study, among 9820 adults (mean age 65.1), each 0.1 mmol/L increase in serum magnesium level was linked to an 18% lower risk of coronary heart disease (CHD) death. Those in the lowest serum magnesium quartile had a 36% higher CHD mortality risk and 54% higher SCD risk. Low magnesium levels were also tied to faster subclinical atherosclerosis and longer QT intervals [223].

Low magnesium levels are confirmed causes of arrhythmias and sudden death—fatal events more common in athletes than in the general population. It is essential to prevent magnesium deficiency and implement supplementation, if necessary, due to the increased requirements in athletes.

#### 3.3.4. Respiratory System

Bronchial asthma, often seen in athletes, involves airway narrowing and inflammation. Respiratory issues, especially exercise-induced bronchoconstriction, are more common in elite athletes (20–70% prevalence), with risk influenced by sport type, environment, and genetics [224].

Magnesium deficiency, both in serum and cells, is common in asthma patients [225,226,227], exacerbating asthma symptoms [227,228,229,230]. Magnesium helps to reduce airway constriction through calcium antagonism, reduced neutrophil activity, and cAMP modulation [231,232]. Low levels may worsen asthma by impairing neuromuscular function and promoting bronchospasm [231].

Magnesium has been used to treat asthma since 1936 and is effective via IV or inhalation in improving lung function and bronchodilation [225,227,229,233,234,235,236]. The MAGNETIC trial showed lasting benefits in children [237]. Magnesium may also modulate immune responses and reduce asthma-related cytokines [238]. A recent review found that two-thirds of studies reported that magnesium sulfate treatment improved asthma exacerbation, assessed by FEV(1)/PEF [239]. Magnesium is essential for vitamin D metabolism (see below), and deficiency in both can worsen airway reactivity and infection risk [37,240].

The possibility that magnesium can prevent and treat asthma attacks has been studied. There are no good-quality data in athletes, although this population could benefit, given their higher risk of this disease.

## 4. Vitamin D and Magnesium Interactions

**Figure 3:**
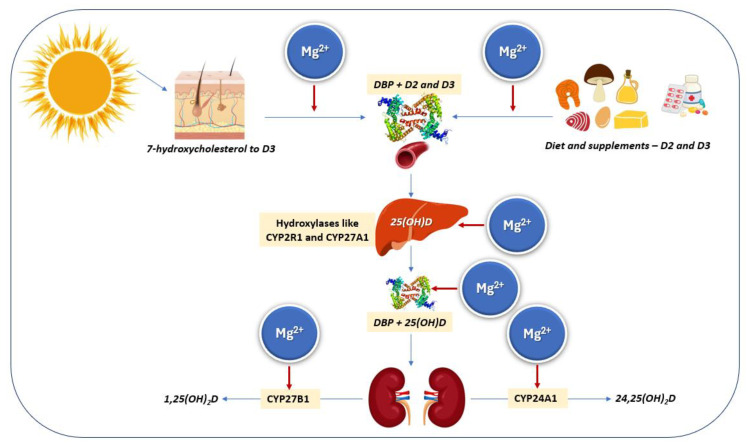
Magnesium is required in the transport and activation of vitamin D. Vitamin D3 is produced in the skin via UVB exposure acting on 7-dehydrocholesterol, followed by a heat-driven reaction. Both skin- and diet-derived vitamin D (D3—cholecalciferol and D2—ergocalciferol, respectively) are transported by DBP and converted in the liver to 25(OH)D (calcifediol), then further metabolized in the kidneys (and other organs as needed) to the active hormone 1,25(OH)_2_D (calcitriol). Magnesium serves as a cofactor for D2 and D3 binding to DBP, hepatic 25-hydroxylation, 25(OH)D transport, and renal 1α-hydroxylation and 24-hydroxylation. Thus, all steps in vitamin D metabolism are magnesium dependent. CYP: Cytochrome P450; DBP: vitamin D binding protein.

Notably, magnesium and vitamin D are closely linked—vitamin D aids magnesium absorption, while magnesium is essential for vitamin D synthesis, transport, and activation (Figure 3). Deficiency in magnesium can impair both vitamin D metabolism and function [37].

In addition, magnesium is vital for PTH synthesis and secretion, and low magnesium levels can impair PTH and vitamin D function, creating a cycle that worsens both deficiencies [241,242,243,244,245]. This dual deficiency of vitamin D and magnesium is linked to increased risk of fractures, especially in women [206]. This harmful interaction may contribute to other health issues. Deng et al. found that higher magnesium intake was linked to a lower risk of vitamin D deficiency and that the inverse link between vitamin D levels and mortality from colorectal cancer and CVD was strongest in those with higher magnesium intake [240]. Similarly, a double-blind RCT from the Personalized Prevention of Colorectal Cancer Trial showed that optimal magnesium levels improved 25(OH)D levels, confirming magnesium’s role in vitamin D metabolism [38]. Another study found that combined vitamin D and magnesium deficiencies were linked to greater asthma severity and more frequent exacerbations [226].

In a study of 192 British Olympic and Paralympic athletes, 22% showed cellular magnesium deficiency, particularly among women, Black/Mixed ethnicity athletes, and those with tendon pain, while higher levels were seen in throwers and Paralympians with cerebral palsy [175].

Magnesium absorption declines with vitamin D deficiency [246], which is common in athletes (see Section 2.2), especially Black athletes [247] due to reduced vitamin D synthesis in darker skin [248]. Pollock et al. found lower red cell magnesium levels in Black and Mixed Race athletes and in women [247], mirroring vitamin D deficiency trends [51]. These findings suggest the need to monitor magnesium status in athletes with low vitamin D levels [38].

Thus, magnesium and vitamin D are crucial for athletes, supporting energy, immunity, and musculoskeletal health. Due to increased exercise demands, they may be prone to magnesium and vitamin D deficiencies. Table 7 outlines key metabolic pathways affected by magnesium and vitamin D in athletes.

Vitamin D intake is generally recommended in the range of 600 to 2000 IU/day by international health agencies [6], although the optimal dose should be tailored to individual status, best determined through blood testing. Athletes may be at higher risk of deficiency due to factors such as indoor training (e.g., swimmers, gymnasts), darker skin tone, living at high latitudes or during winter, and limited sun exposure from clothing or sunscreen use. Blood levels of 25(OH)D should ideally be maintained between 30 and 50 ng/mL. Toxicity is a concern when concentrations exceed 100 ng/mL, particularly in those consuming high levels of calcium [64].

The recommended daily magnesium intake is 420 mg for men and 320 mg for women in the U.S. adult population [249] and 350 mg for men and 300 mg for women in Europe [250]. Athletes may require 10–20% more due to increased losses (sweat, urine), higher metabolic demands, and suboptimal dietary intake [29]. Key food sources include green vegetables, whole grains, legumes, pseudocereals, nuts, seeds, and cocoa, avoiding conditions that may decrease bioavailability (Table 4). Magnesium supplements (200–400 mg/day of elemental magnesium) are recommended when intake is insufficient or deficiency symptoms persist. Although the signs of magnesium deficiency are often nonspecific [11], suboptimal magnesium levels should be considered when persistent symptoms such as muscle cramps or spasms, fatigue, low endurance, irritability, poor sleep, delayed recovery, or increased injury risk are present. The serum magnesium level may not reflect cellular levels, but it will exclude severe deficiency [119].

## 5. Conclusions

Vitamin D and magnesium are essential elements for preserving the health of athletes due to their key effects on musculoskeletal, cardiovascular, and respiratory functions. These elements are also essential for supporting athletic performance. Deficiencies in both of these crucial compounds are common among athletes and can compromise their training and physical performance, promote injuries (e.g., stress fractures), arrhythmias, sudden cardiac death, asthma, and infections, as well as hinder proper recovery.

The evidence reviewed suggests that most athletes fail to meet the recommended magnesium intake through diet alone and that they have higher requirements. Athletes are also prone to vitamin D deficiency. There are no data on the presence of both deficiencies simultaneously. There is also no definitive evidence on the possibility that supplementation with these nutrients could have any effect on athletic performance, and there are no data on the effects of their combination, given that they are closely related and mutually necessary compounds. However, despite the lack of specific studies on these outcomes, it is clear and prudent to ensure that athletes do not have deficiencies in these two fundamental nutrients due to the relevant consequences, including stress fractures, sudden cardiac death, and infections.

One-size-fits-all strategies, high dose or bolus administration, and overlooking personalized protocols are not recommended. Instead, it is necessary to attentively evaluate and monitor the statuses of vitamin D and magnesium, advise following an adequate diet and sun exposure throughout the year, and if necessary, use supplements to obtain and maintain adequate levels of the two essential compounds for athletes’ health.

## Figures and Tables

**Table 1:** Dietary sources of vitamin D.

Food	Serving	IU Per Serving
Cod liver oil	1 tablespoon	1360
Trout (cooked)	3 ounces	645
Salmon (cooked)	3 ounces	570
Mushrooms (raw, exposed to UV light)	1/2 cup	366
Sardines	2 sardines	46
Egg	1 large	44
Liver, beef (braised)	3 ounces	42
Tuna fish (canned, drained)	3 ounces	40
Cheese, cheddar,	1 ounce	12
Mushrooms (raw)	1/2 cup	4
Chicken breast (roasted)	3 ounces	4
Beef (ground, lean, broiled)	3 ounces	1.7

IU: international units. Data from Vitamin D Fact Sheet for Health Professionals, National Institute of Health (https://ods.od.nih.gov/factsheets/VitaminD-HealthProfessional/#h3, accessed on 29 March 2025).

**Table 2:** Most common vitamin D metabolites, their sites of production, and the enzymes involved.

Metabolite	Chemical Formula	Site of Production	Enzyme
7-Dehydrocholesterol	** 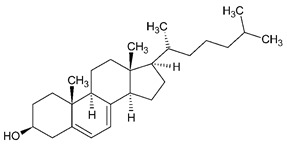 **	Skin	Lathosterol oxidase
Vitamin D3 (cholecalciferol)	** 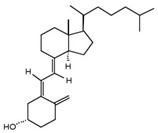 **	Skin or diet (animal origin)	CYP27A1 and CYP27B1
Vitamin D2 (ergocalciferol)	** 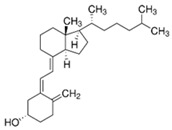 **	Diet (vegetable origin)	
25(OH)D (Calcifediol or calcidiol)	** 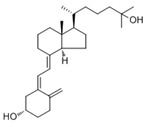 **	Liver	Hydroxylases like CYP2R1 and CYP27A1
1,25 (OH)_2_D (Calcitriol)	** 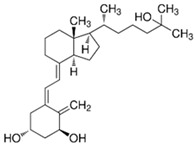 **	Kidney	CYP27B1
24,25 (OH)_2_D	** 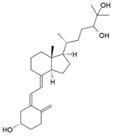 **	Most tissues	CYP24A1

Abbreviations: CYP: Cytochrome P450.

**Table 3:** Magnesium exerts multiple fundamental functions for cell function.

Cellular Functions of Magnesium
Cofactor for over 600 enzymes Ca^2+^ channel blocker Energy compound synthesis (ATP, ADP) ↓ Oxidative stress ↓ Chronic inflammation NMDA receptor blocker RNA transcription protein synthesis DNA replication, stability, repair All phosphorylation/dephosphorylation reactions

Abbreviations: ADP: adenosine diphosphate; ATP: adenosine triphosphate; DNA: deoxyribonucleic acid; NMDA: N-methyl-D-aspartate receptor; RNA: ribonucleic acid; ↓: low.

**Table 4:** Primary dietary sources of magnesium and factors that affect its bioavailability.

Food	↑ Bioavailability	↓ Bioavailability
Green vegetables Whole cereals Legumes Pseudocereals Nuts and seeds Cocoa	Vitamin D and B6 Fermentable soluble fiber Unprocessed food Whey proteins Magnesium-rich water	Alcohol Ultra-processed and processed food Excessive calcium intake Excessive coffee consumption Soft drinks Some medications

↑: high; ↓: low.

**Table 5:** Effects of magnesium on muscle.

Magnesium Effects on Muscle
Participates in muscle contraction Essential for protein synthesis Essential for ATP synthesis and utilization Supports core mitochondrial functions ↓ Oxidative stress ↓ Inflammation Regulates electrolyte balance

Abbreviations: ATP: adenosine triphosphate; ↓: low.

**Table 6:** Effects of magnesium on cardiovascular tissues.

Magnesium Effects on Cardiovascular System
Coronary vasodilation Calcium antagonist Improvement in endothelial dysfunction Improvement in glucose and insulin metabolism Production and activation of ATP (myocardial contraction and mitochondrial oxidative phosphorylation) Regulation of other ions via Na/K ATPase Reduced platelet aggregation Antioxidant and anti-inflammatory effects Prevention and treatment of arrhythmias Prevention of vascular calcifications

Abbreviations: ATP: adenosine triphosphate.

**Table 7:** Flowcharts summarizing the main metabolic pathways influenced by magnesium and vitamin D in athletes.

Magnesium	Vitamin D
1. Energy Production (ATP Synthesis) Magnesium-ATP complex: Required for ATP stability and utilization Glycolysis: Magnesium activates hexokinase, phosphofructokinase TCA Cycle: Cofactor for enzymes like isocitrate dehydrogenase Oxidative Phosphorylation: Supports electron transport chain efficiency	1. Calcium, Phosphorus & Magnesium Homeostasis Vitamin D increases intestinal absorption of calcium, phosphorus, and magnesium Enhances bone mineralization and strength Supports muscle contraction (via calcium regulation)
2. Muscle Function & Contraction Magnesium regulates calcium flow in muscle cells Balances muscle contraction and relaxation	2. Muscle Function & Strength VDRs in muscle tissue Stimulates protein synthesis Improves muscle fiber contraction and coordination
3. Protein Synthesis Magnesium is essential for ribosomal stability, mRNA translation Supports muscle repair and growth post-exercise	3. Bone & Injuries Bone mineralization Enhances recovery and reduces risk of injury (stress fractures)
4. Glucose Metabolism Magnesium is necessary for insulin signaling Improves glucose uptake into cells Enhances glycogen synthesis in liver and muscle Maintains blood sugar levels during/after exercise	4. Immune Modulation Vitamin D modulates innate and adaptive immunity Reduces inflammation May lower risk of respiratory infections in athletes Supports recovery from intense training
5. Electrolyte Balance & Nerve Transmission Calcium, potassium, sodium Regulates nerve impulses Prevents cardiac arrhythmias and sudden death	5. Endocrine Regulation May aid muscle growth and endurance Affects energy metabolism and glucose regulation
